# Integrative computational approach identifies immune‐relevant biomarkers in ulcerative colitis

**DOI:** 10.1002/2211-5463.13357

**Published:** 2022-01-12

**Authors:** Tianzhen He, Kai Wang, Peng Zhao, Guanqun Zhu, Xinbao Yin, Yulian Zhang, Zongliang Zhang, Kai Zhao, Zhenlin Wang, Ke Wang

**Affiliations:** ^1^ Institute of Special Environmental Medicine Nantong University China; ^2^ Key Laboratory of Epigenetics and Oncology The Research Center for Preclinical Medicine Southwest Medical University Luzhou China; ^3^ Faculty of Sport Science and Coaching Universiti Pendidikan Sultan Idris Tanjong Malim Malaysia; ^4^ Athletics Department Duke Kunshan University China; ^5^ Department of Urology the Affiliated Hospital of Qingdao University Qingdao University China; ^6^ Department of Gynecology the Affiliated Hospital of Qingdao University Qingdao University China

**Keywords:** bioassay, computational approach, immune‐related biomarkers, leukocyte migration, ulcerative colitis

## Abstract

Ulcerative colitis is a common inflammatory bowel disease with a complex genetic and immune etiology. Immune infiltration plays a vital role in the development of ulcerative colitis. To explore potential biomarkers for ulcerative colitis and analyze characteristics of immune cell infiltration, we used bioinformatic analyses, including machine learning algorithms, cell type deconvolution methods, and pathway enrichment methods. In this study, we identified 216 differentially expressed mRNAs (DEMs), of which 153 were upregulated, and 63 were downregulated genes. DEMs were mainly enriched in infiltrating neutrophils and regulation of leukocyte migration. Moreover, eight candidate biomarkers, DPP10, MST1L, DPP10‐AS1, CEP55, ACSL1, MGP, OLFM4, and SGK1, were identified. Of these candidate biomarkers, MST1L, OLFM4, and DPP10 were then validated in the GSE48958 dataset and were predicted to be strongly correlated with infiltrating immune cells of ulcerative colitis. The underlying mechanism of these key genes in the development of colitis was also predicted by gene set variation analysis. To further validate these biomarkers' expression in ulcerative colitis, we determined mRNA levels of *SGK1*, *CEP55*, *ACSL1*, *OLFM4*, and *DPP10* in lipopolysaccharides (LPS)‐stimulated Raw264.7 cells by quantitative reverse transcription‐polymerase chain reaction. We also examined *SGK1*, *CEP55*, *ACSL1*, *OLFM4*, *DPP10*, and *MGP* expression in the colon tissues of dextran sodium sulfate‐induced colitis mice. Consistent with the predicted computational results, the mRNA levels of these candidate genes were markedly changed in LPS‐stimulated Raw264.7 cells and inflamed colon tissues. Hence, our findings indicated that these critical genes may act as diagnostic biomarkers for ulcerative colitis and that differential immune infiltration cells may help illustrate the progression of ulcerative colitis.

## Graphical abstract

In this study, we identified eight critical genes closely related to ulcerative colitis by machine learning algorithms and validated them in GSE48958 and quantitative reverse transcription‐polymerase chain reaction in *in vitro* bioassays. For these genes, we also analyzed their characteristics of immune cell infiltration and their downstream signaling pathways. Hence, our findings indicated that these genes may act as diagnostic biomarkers for ulcerative colitis.

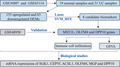

**Abbreviations**
AUC: area under the curve
DEMs: differentially expressed mRNAs
DSS: dextran sodium sulfate
ES: enrichment score
GEO: Gene Expression Omnibus
GO: Gene Ontology
GSVA: gene set variation analysis
KEGG: Kyoto Encyclopedia of Genes and Genomes
LPS: lipopolysaccharides
PCA: principal component analysis
PPI: protein–protein interaction
qPCR: quantitative reverse transcription‐polymerase chain reaction
SVM_RFE: support vector machine recursive feature elimination
WT: wild‐type
γδ T cells: gamma‐delta T cells

Ulcerative colitis, a type of inflammatory bowel diseases, is characterized by chronic inflammation of the colon, in which the lining of the colon becomes inflamed and develops tiny open sores or ulcers [1]. Recently, the occurrence of ulcerative colitis has increased all over the world with nearly 0.5–31.5 cases per 100 000 persons are at risk of ulcerative colitis [2]. The disease has long‐term severe local and systemic consequences and often recurs. Currently, the diagnosis and treatment of ulcerative colitis are still limited by existing technologies. Ulcerative colitis diagnosis largely relies on gastrointestinal endoscopy and mucosal histopathological biopsy, which will delay the effective therapeutic opportunity of some ulcerative colitis patients with atypical endoscopic signs or pathological features [3]. At the same time, the treatment of ulcerative colitis includes both medical and surgical therapies. Generally, patients with mild‐to‐moderate ulcerative colitis without complications do not need surgical treatment but are administered drugs such as glucocorticoids [4]. However, it is worth noting that ineffective treatment or drug resistance, or severe side effects are accompanied. Therefore, the exploration of diagnostic biomarkers and therapeutic targets is urgently necessary to improve the outcomes of ulcerative colitis.

To identify new therapeutic targets for the diagnosis and treatment of ulcerative colitis, it is crucial to further understand the pathophysiology of disease. The pathophysiology of ulcerative colitis is quite complex, involving at least three different components: the immune system, epithelial barrier function, and intestinal flora [5, 6]. The host immune system represents the main effector of the inflammatory response in ulcerative colitis. It has been reported that the NLRP3 inflammasome was recruited to promote the development of ulcerative colitis by increasing the secretion of pro‐inflammatory cytokines [7]. In addition, IRF5 induced the inflammatory response by regulating T‐cell signaling and cytokine production [8]. The neutrophilic HGF‐MET axis was also involved in promoting the progression of ulcerative colitis [9]. Then, sphingolipids were reported to regulate the inflammatory response in ulcerative colitis by modulating neutrophil function [10]. Increasing evidence shows that the level of immune infiltration analysis is closely associated with clinical outcomes [11], and these infiltrated immune cells were involved in excessive inflammation of mucosal tissues [12]. These findings demonstrated the key role of immune cells in the pathogenesis of ulcerative colitis. Molecules associated with these immune cells may serve as new biomarkers of ulcerative colitis.

In our present study, by using this bioinformatic method, we first investigated a list of critical genes closely related to ulcerative colitis by Lasso and SVM_RFE and validated them in the GSE48958 dataset. Furthermore, the correlation between these critical genes and immune cells was analyzed by CIBERSORT. The potential regulatory pathways of these candidate genes were also identified by gene set variation analysis (GSVA). To further validate the expression of these candidate biomarkers in ulcerative colitis, we determined mRNA levels of *SGK1*, *CEP55*, *ACSL1*, *OLFM4*, and *DPP10* in lipopolysaccharides (LPS)‐stimulated Raw264.7 cells by quantitative reverse transcription‐polymerase chain reaction (qPCR) in *in vitro* bioassays. In addition, we also examined the expression of *SGK1*, *CEP55*, *ACSL1*, *OLFM4*, *DPP10*, and *MGP* in the colon tissues of dextran sulfate sodium‐induced colitis mice. Consistent with the predicted computational results, the mRNA levels of these candidate genes were markedly changed in LPS‐stimulated Raw264.7 cells and inflamed colon tissues. Taken together, the integrated analysis of immune infiltration cells and immune‐related genes provided new biomarkers for the diagnosis of ulcerative colitis.

## Methods

### Dataset sources

The series matrix file data were downloaded from the GSE36807 of NCBI Gene Expression Omnibus (GEO) public database (https://www.ncbi.nlm.nih.gov/geo/) [13], including 22 sets of microarray data, of which 7 cases were divided into the normal group and 15 cases were in the ulcerative colitis group. The matrix file data from the GSE65114 were also downloaded, which contained 28 sets of samples, of which 12 cases were in the normal group, 16 cases in the ulcerative colitis group (these ulcerative colitis data were singled out from that of Crohn's disease patients). SVA algorithm was used to normalize these data, and the limma package was used to identify the differentially expressed genes. The criteria for identifying differentially expressed genes were as follows: |logFC| > 1 and *P* < 0.05. To further verify the expression profiles of key genes, the series matrix file data were downloaded from the GSE48958 dataset, which has 21 sets of microarray data, including 8 cases in the normal group and 13 cases in the colitis group.

### Feature selection with Lasso and SVM_RFE

For feature selection of ulcerative colitis diagnostic markers, we used Lasso logistic regression [14] and support vector machine recursive feature elimination (SVM**_**RFE) [15]. The Lasso algorithm uses the ‘glmnet’ software package, the response type was setting as binomial, and the alpha was identified as 1. In addition, SVM**_**RFE is a machine learning method based on support vector machines. It searches for the best variables by deleting the feature vectors generated by SVM and establishes a support vector machine model through the ‘e1071’ software package to further identify these biomarkers with diagnostic value in ulcerative colitis, the *k* = 5 was chosen for the *k*‐fold cross‐validation, and the parameter of halve.above was setting as 100.

### Biological function enrichment analysis

To obtain the biological functions and signaling pathways involved in the differential genes, we used the Metascape database (www.metascape.org) for annotation and visualization, Gene Ontology (GO) for differential gene analysis, and Kyoto Encyclopedia of Genes and Genomes (KEGG) for pathway analysis [16]. The adjusted *P*‐value < 0.05 was set as the cutoff criteria.

### Immune infiltration analysis

The CIBERSORT algorithm (https://cibersort.stanford.edu/) was used to analyze the expression profile data of the normal group and the ulcerative colitis group from the combined dataset of GSE36807 and GSE65114, and infer the relative proportion of 22 immune infiltrating cells in the inflamed colon tissues [17]. To explore the distribution of immune cells among different subgroups, we used the ‘pheatmap’ package to draw immune cell infiltration heat maps. Furthermore, we also used the ‘corrplot’ package to analyze the interaction of different immune cells and the ‘vioplot’ package to plot the relative proportion of immune cells. *P* < 0.05 was considered statistically different.

### Gene set variation analysis

Gene set variation analysis is a nonparametric and unsupervised method which estimates the relative enrichment of a gene set of interest across a sample population [18]. Hence, it allows us to observe the variation in the activity of a set of genes, such as a pathway or a gene signature, corresponding to a particular biological condition, within an entire gene expression set. It produces a value, termed enrichment score (ES), per sample and gene set, which can be examined for associations with clinical features of interest. In this study, gene sets from the Molecular signatures database (v7.0 version) were downloaded, and the GSVA algorithm was used to comprehensively score each gene set to evaluate the potential biological function changes in different samples.

### Cell culture and RNA isolation

To determine the fold change in the *SGK1*, *CEP55*, and *ACSL1* mRNA expression by macrophages, we plated Raw264.7 cells (1 × 10^6^ cells) onto 24‐well tissue culture plates and treated by 100 ng·mL^−1^ of LPS (Sigma‐Aldrich, Cat#: L9764, Hong Kong SAR, China) for 24 h, and cells were then harvested to determine the mRNA level by qPCR. Total RNA extraction was then performed according to the manufacturer's protocol (Qiagen RNeasy mini kit, Germantown, MD, USA). The extracted RNA was quantified by absorbance at 260 nm and the absorbance ratio evaluated the purity at 260/280 nm with a NanoDrop ND‐100 spectrophotometer (NanoDrop Technologies, Wilmington, DE, USA).

### DSS‐induced ulcerative colitis

Female wild‐type (WT) C57BL/6J (6–8 weeks old) were provided by the Animal Facility of Southwest Medical University. These mice were housed at 4–5 mice to a cage on a 12‐h light/12‐h dark cycle, and mice had *ad libitum* access to food and water. The animal study protocol was approved by Animal Research Ethics Committee of Southwest Medical University. To induce acute ulcerative colitis, we followed the previous protocol with minor modifications [19]. Here, 6‐ to 8‐week‐old mice were given drinking water containing 2.5% (w/v) dextran sodium sulfate (DSS; MP Biomedicals) *ad libitum* for 7 days and distilled water for one additional day before sacrifice. Mice were monitored daily for clinical signs, including weight loss, mobility, and fecal bleeding. All the mice were anesthetized before sacrifice. The colon tissues (from cecum to rectum) were separated from sacrificing mice. Colon length was recorded. Then, colon tissues were collected for the measurement of these candidate gene expressions by qPCR and for histological assessment of inflammatory infiltration.\

### Quantitative reverse transcription‐polymerase chain reaction

Total RNA was extracted as described above. qPCR was performed using a Qiagen RT‐PCR kit with SYBR Green and specific primers. GAPDH‐specific primer served as internal controls. qPCR primer sequences were as follows: Mouse *SGK1* forward primer CTGCTCGAAGCACCCTTACC; reverse primer TCCTGAGGATGGGACATTTTCA. Mouse *CEP55* forward primer CCTAGTAGCTCCAAGTCAGACA; reverse primer ACCTTAGGTGGTCTTTGAGTCTC. Mouse *ACSL1* forward primer TGCCAGAGCTGATTGACATTC; reverse primer GGCATACCAGAAGGTGGTGAG. Mouse *MGP* forward primer GGCAACCCTGTGCTACGAAT; reverse primer CCTGGACTCTCTTTTGGGCTTTA. Mouse *OLFM4* forward primer ACGGTCCGAGTAGAGGTCAT; reverse primer CCATGACTACAGCTTCCAGGAG. Mouse *DPP10* forward primer CGAGGAAGTGTGAGCTCCG; reverse primer TACTTCCTAGTTCCTGGTCGG. *GAPDH* forward primer AAATGGTGAAGGTCGGTGTGAAC; reverse primer CAACAATCTCCACTTTGCCACTG.

### Statistical analysis

For bioinformatic analysis, all statistical analyses were two‐side tests and performed by using r language (version 3.6, R Foundation for Statistical Computing, Vienna, Austria). For *in vitro* bioassay, comparisons of two groups of data were analyzed by two‐tailed Student's *t*‐test by using graphpad prism 7.0. (GraphPad, San Diego, CA, USA). All *P*‐values less than 0.05 were considered significant.

### Ethics approval

Reusable datasets for our analysis comply with relevant ethical regulations. The animal study protocol was approved by Animal Research Ethics Committee of Southwest Medical University.

## Results

### Identification of differentially expressed mRNAs in inflamed colons

**Fig. 1:**
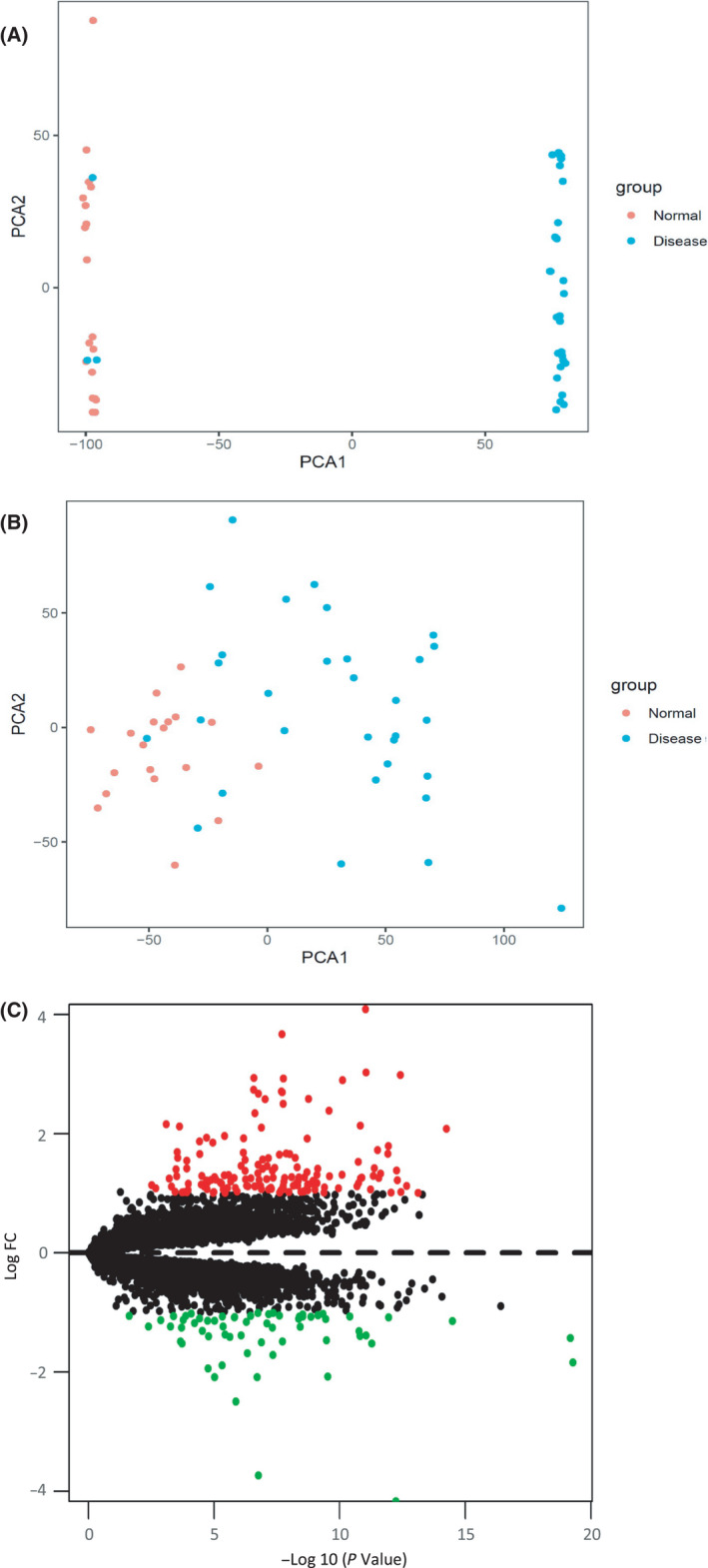
Data collection, processing, and DEMs in inflamed colons were identified. (A, B) PCA cluster plots for the merged gene expression matrix before and after normalized by SVA methods in the datasets of GSE36807 and GSE65114. Each point represented a sample. (C) Volcano plots of DEMs were shown. Black dots represent genes equally represented between ulcerative colitis and normal samples. Red and green dots represent upregulated and downregulated mRNAs, respectively. Volcano plots showed all DEMs identified from the combined data of GSE36807 and GSE65114 by limma after SVA algorithm normalization. |log2FC| > 1 and *P* < 0.05 were set as cutoff criteria.

To identify differentially expressed mRNAs (DEMs) in ulcerative colitis, we downloaded data from two independent mRNA expression arrays (GSE36807 and GSE65114) from the GEO public database, including 22 samples in GSE36807 (including 7 healthy controls and 15 ulcerative colitis) and 28 samples in GSE65114 (including 12 healthy controls and 16 ulcerative colitis). The two datasets, GSE36807 and GSE65114, were combined and analyzed by the limma software package of R language. There were obvious batch effects between the two datasets (Fig. 1A). The merged gene expression matrix was normalized by using SVA methods, and the two‐dimensional principal component analysis (PCA) cluster plot showed that the batch effects had been removed (Fig. 1B). These suggested that the normalized data from these datasets were suitable for subsequent analyses. A total of 216 DEMs were found in GSE36807 and GSE65114 datasets according to the thresholds set (*P* < 0.05 and |log2FC| > 1), including 153 upregulated DEMs and 63 downregulated DEMs (Fig. 1C). Then, we showed the top 20 DEMs in these combined data in Table 1.

**Table 1:** Top 20 differently expressed mRNAs in inflamed colons (GSE36807 and GSE65114). All statistical analyses were two‐side tests and performed by using r language (version 3.6).

Gene symbols	Log FC	Ave expr	*P*. value	adj. *P*. value
MMP1	4.08789282	7.286392302	9.22E‐12	2.10E‐09
REG1A	3.667185364	7.100532429	1.97E‐08	6.34E‐07
SFRP2	3.024466262	6.242897462	8.81E‐12	2.10E‐09
S100A8	2.984505645	6.586651892	3.81E‐13	2.58E‐10
DUOX2	2.933674449	8.532211166	2.56E‐07	4.44E‐06
DMBT1	2.923223282	9.543393812	1.71E‐08	5.74E‐07
S100A12	2.898608483	5.406117736	7.61E‐11	9.99E‐09
CXCL9	2.737407746	8.130534633	2.66E‐07	4.60E‐06
*DUOXA2*	2.708580477	7.056895918	2.06E‐08	6.60E‐07
*CHI3L1*	2.693707511	6.506399349	1.95E‐08	6.31E‐07
*CLDN8*	−4.172343588	6.295416945	5.77E‐13	2.94E‐10
*AQP8*	−3.735311254	9.016016773	1.69E‐07	3.22E‐06
*PCK1*	−2.498494655	7.768940184	1.33E‐06	1.68E‐05
*HMGCS2*	−2.08625539	9.242639176	9.56E‐06	8.02E‐05
*CHP2*	−2.08456621	8.798136748	1.91E‐07	3.51E‐06
*CNTN3*	−2.074178731	5.799165058	2.96E‐10	2.89E‐08
*GUCA2A*	−1.94378994	10.31210859	1.72E‐05	1.30E‐04
*PNLIPRP2*	−1.886205694	5.943337142	4.81E‐06	4.56E‐05
*DPP10*	−1.841553271	4.520526925	5.18E‐20	3.99E‐16
*CA1*	−1.714709703	6.993235628	4.50E‐08	1.20E‐06

### The function of these DEMs may be associated mainly with immune regulation

**Fig. 2:**
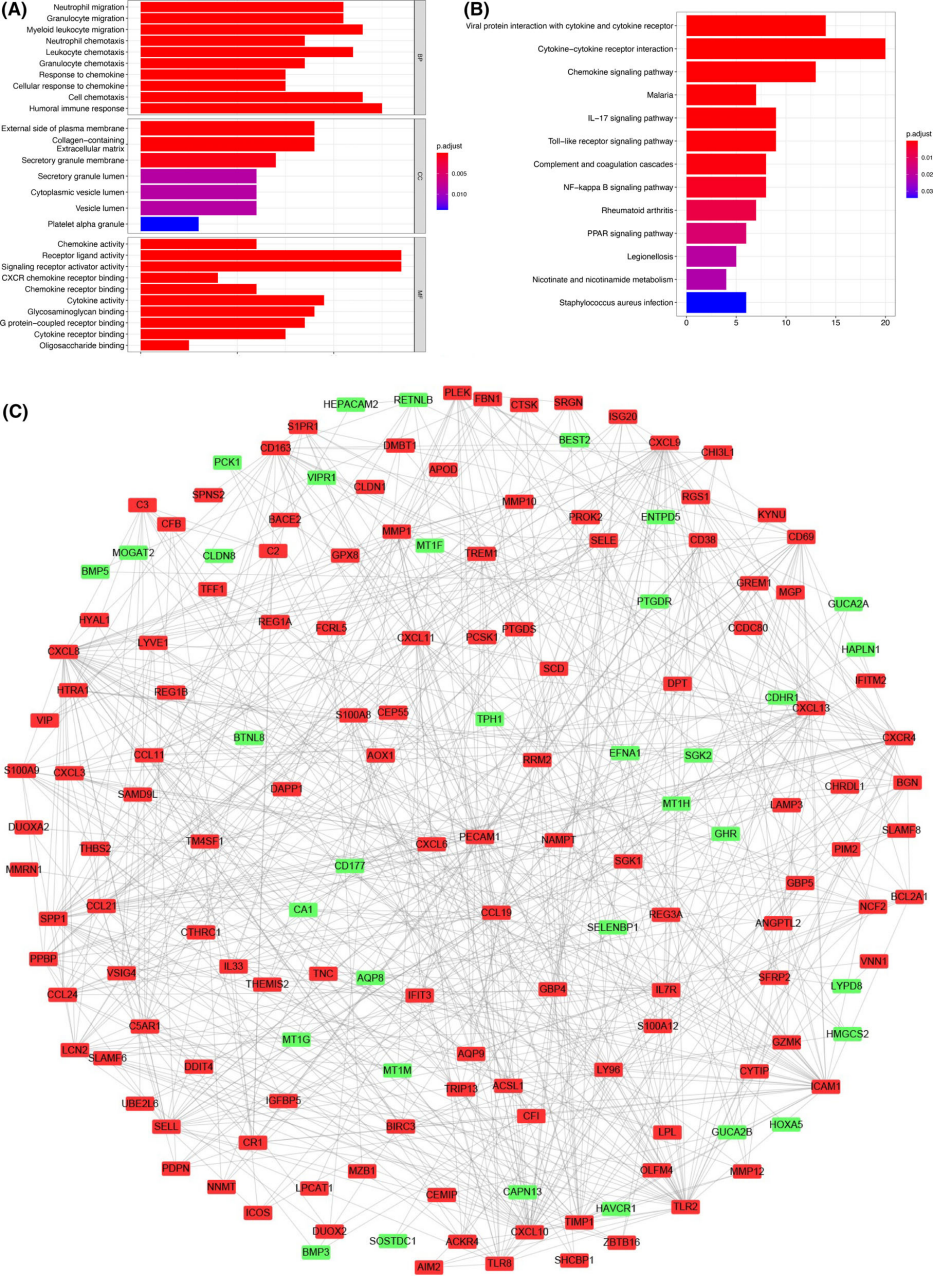
GO and KEGG analysis and PPI networks of differently expressed mRNAs. (A, B) GO and KEGG analysis of all differently expressed mRNAs. (C) PPI network analysis of differently expressed mRNAs.

To investigate the biological function of these DEMs in the development of ulcerative colitis, GO function and KEGG pathway enrichment analysis were performed by the Metascape database. The results of GO terms for biological processes showed that the most enriched terms were ‘neutrophil migration’, ‘granulocyte migration’, ‘myeloid leukocyte migration’, ‘neutrophil chemotaxis’ and ‘leukocyte chemotaxis’ (Fig. 2A). The enriched GO terms for cell components of DEMs included ‘external side of plasma membrane’, ‘collagen‐containing extracellular matrix’ and ‘secretory granule membrane’ (Fig. 2A). In addition, enriched GO terms for molecular function also revealed that DEMs were mainly involved in immune‐related processes such as chemokine activity and chemokine receptor binding (Fig. 2A). KEGG pathway enrichment analysis revealed the DEMs were mostly enriched in terms of immune signaling pathways, including chemokine signaling pathway, IL‐17 signaling pathway, and Toll‐like receptor signaling pathway, which were involved in the development of ulcerative colitis (Fig. 2B). In addition, the function of DEMs at the protein level shown by the protein–protein interaction (PPI) network was also mainly associated with immune regulation and response in Fig. 2C. Therefore, these results indicated that immune signaling pathways may be involved in the pathophysiology of ulcerative colitis.

### Candidate biomarkers in ulcerative colitis were identified and validated

**Fig. 3:**
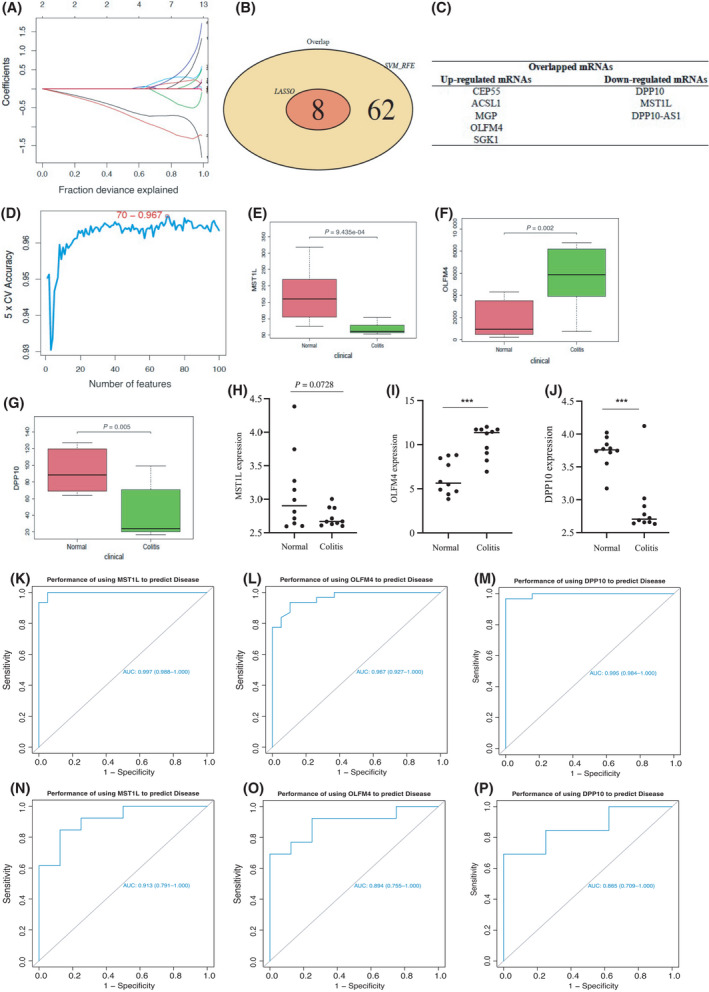
Key genes were identified by Lasso and SVM_RFE. (A) Lasso plot. (B) Venn diagram of Lasso and SVM_RFE. (C) The summary of overlapped mRNAs by Lasso and SVM_RFE. (D) SVM accuracy. (E–G) The expression of crucial genes *MST1L*, *OLFM4*, and *DPP10* was validated in the GSE48958 dataset. (H–J) The expression profiling of *MST1L*, *OLFM4*, and *DPP10* in human colon in GDS4519. (K–M) ROC analysis of *MST1L*, *OLFM4*, and *DPP10* was performed on the combined dataset of GSE36807 and GSE65114. (N–P) ROC analysis of *MST1L*, *OLFM4*, and *DPP10* genes was performed in the GSE48958 dataset. Compared with the indicated group, ***P < 0.001. All statistical analyses were two‐side tests and performed by using r language (version 3.6).

Two independent algorithms, Lasso and SVM_RFE, were used to screen and identify candidate biomarkers among these DEMs. The lasso method showed that eight genes were identified as potential biomarkers of ulcerative colitis, while the SVM_RFE showed that 70 genes were potential signatures of ulcerative colitis. As a result, eight overlapped candidate biomarkers were identified, including DPP10, MST1L, DPP10‐AS1, CEP55, ACSL1, MGP, OLFM4, and SGK1 (Fig. 3A–C). The results of 5× CV accuracy showed that the accuracy of SVM algorithm was very high (Fig. 3D). To further confirm the reliability and reproducibility of these results, we validated the expression of critical genes *MST1L*, *OLFM4*, and *DPP10* in the GSE48958 dataset. As a result, the expression levels of *MST1L*, *OLFM4*, and *DPP10* in GSE48958 were consistent with that of the combined dataset of GSE36807 and GSE65114 (Fig. 3E–G). The same expression profiling of *MST1L*, *OLFM4*, and *DPP10* was obtained in GDS4519 (Fig. 3H–J). Furthermore, to examine the model's accuracy in distinguishing between the healthy and ulcerative colitis groups, we further determine the area under the curve (AUC) of *MST1L*, *OLFM4*, and *DPP10* in GSE48958 and the combined data from GSE36807 and GSE65114. AUC was higher than 0.8, indicating that these genes *MST1L*, *OLFM4*, and *DPP10* are indicators of ulcerative colitis (Fig. 3K–P).

### Expression of *MST1L*, *OLFM4*, and *DPP10* genes was associated with immune cell infiltration in ulcerative colitis

**Fig. 4:**
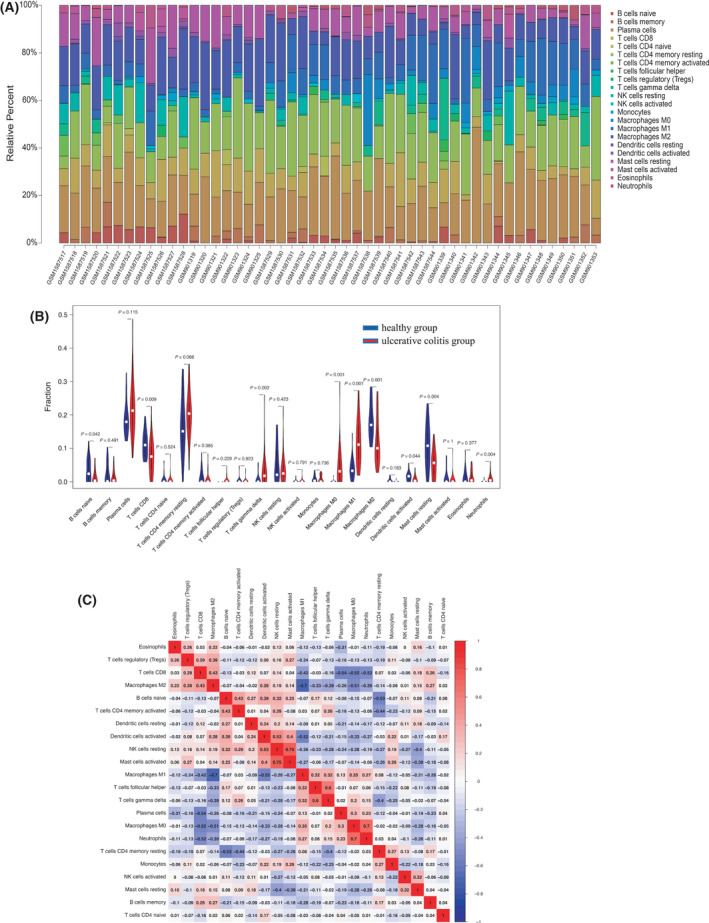
Landscape of immune infiltration between ulcerative colitis and healthy samples from GSE36807 and GSE65114 combined dataset. (A) Bar charts of 22 immune cell proportions in ulcerative colitis and normal samples. (B) Violin plot for the different proportions of infiltrated immune cells between ulcerative colitis and healthy control samples from GSE36807 and GSE65114 combined dataset. The blue and red bar represented the healthy control and ulcerative colitis group, respectively. (C) Correlation matrix of various types of immune cell proportions. All statistical analyses were two‐side tests and performed by using r language (version 3.6). *P*‐value < 0.05 was considered statistically significant.

**Fig. 5:**
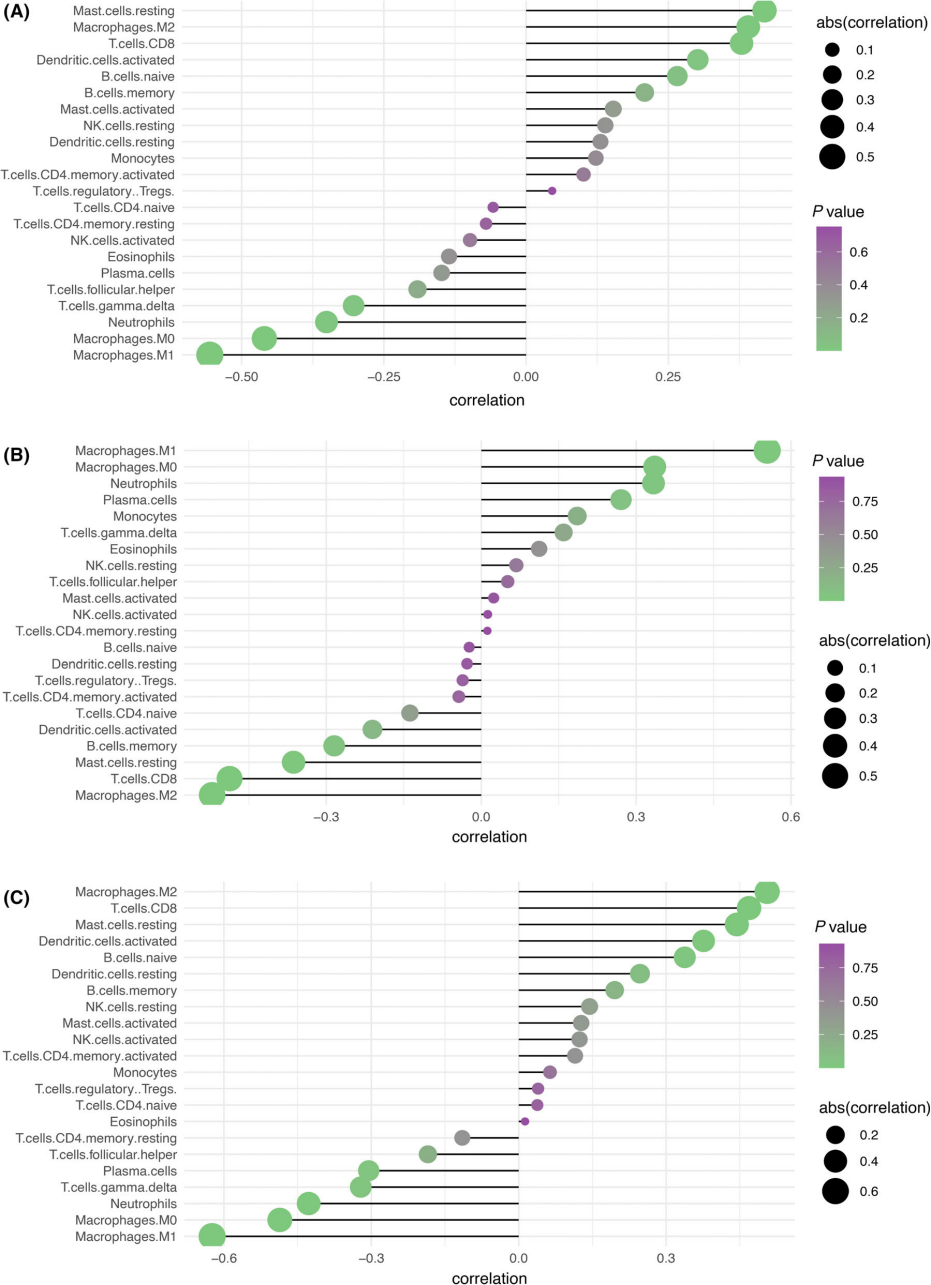
MST1L, OLFM4, and DPP10 were associated with immune infiltration of colitis. (A–C) MST1L, OLFM4, and DPP10 were associated with immune infiltration of colitis, especially T‐cell gamma‐delta, neutrophils, macrophages M0 and M1.

The inflammatory microenvironment mainly contains fibroblasts, immune cells, extracellular matrix, various growth factors, inflammatory factors, and special physical and chemical factors. It is well‐known that the inflammatory microenvironment significantly affects disease diagnosis and clinical treatment sensitivity. To further explore the underlying molecular mechanisms of candidate biomarkers in the progression of ulcerative colitis, the relationship between candidate biomarkers in the dataset and microenvironmental immune cell infiltration was analyzed by R language. In Fig. 4A–C, there are 22 subpopulations of immune cells in ulcerative colitis and healthy control samples from the combined dataset of GSE36807 and GSE65114. The inflamed colon tissues contained higher levels of gamma‐delta (γδ) T cells, neutrophils, and macrophages M1, compared with noninflamed colon tissues. Furthermore, the relationships between candidate ulcerative colitis biomarkers and the immune‐infiltrated cells which differ between ulcerative colitis and normal samples were further calculated with the corrplot package. The results demonstrated that MST1L was negatively correlated with γδ T cells (correlation = −0.303, *P* < 0.05; Fig. 5A and Table 2), neutrophils (correlation = −0.351, *P* < 0.05; Fig. 5A and Table 2), and macrophages M1 (correlation = −0.556, *P* < 0.001; Fig. 5A and Table 2). OLFM4 exhibited positive correlation with neutrophils (correlation = 0.334, *P* < 0.05; Fig. 5B and Table 2) and macrophages M1 (correlation = 0.554, *P* < 0.001; Fig. 5B and Table 2). DPP10 was negatively correlated with γδ T cells (correlation = −0.322, *P* < 0.05; Fig. 5C and Table 2), neutrophils (correlation = −0.428, *P* < 0.01; Fig. 5C and Table 2), and macrophages M1 (correlation = −0.624, *P* < 0.001; Fig. 5C and Table 2). Therefore, these data indicated that MST1L, OLFM4, and DPP10 were strongly associated with infiltrating immune cells in ulcerative colitis.

**Table 2:** Correlation between MST1L, OLFM4, and DPP10 in ulcerative colitis and immune infiltration cells. All statistical analyses were two‐side tests and performed by using r language (version 3.6).

Symbol	MST1L		OLFM4		DPP10	
	Correlation	*P*‐value	Correlation	*P*‐value	Correlation	*P*‐value
B cells naive	0.265776894	0.062111416	0.023352666	0.872114204	0.338007857	0.016361323
B cells memory	0.208358728	0.14648909	−0.284761555	0.045032738	0.195392196	0.173882274
Plasma cells	−0.14839615	0.303720716	0.270720023	0.057231444	−0.305356517	0.031054039
T cells CD8	0.378707783	0.006688613	−0.487410258	0.000330728	0.468872661	0.000593745
T cells CD4 naive	−0.057954865	0.689322976	−0.138063816	0.338993613	0.03788278	0.793945844
T cells CD4 memory resting	−0.070432646	0.62693762	0.011943098	0.934394364	−0.114861784	0.427028542
T cells CD4 memory activated	0.100939015	0.485503422	−0.04342829	0.764590123	0.114783895	0.427344194
T cells follicular helper	−0.190884634	0.18422226	0.05129465	0.72351346	−0.184841015	0.19876733
T cells regulatory Tregs	0.045804616	0.752107759	−0.035909284	0.804463649	0.039411054	0.785825621
T cells gamma delta	−0.303128343	0.032366354	0.159487471	0.268586961	−0.321646483	0.022740967
NK cells resting	0.139261577	0.334779161	0.067921951	0.639302012	0.144302825	0.317401437
NK cells activated	−0.098510752	0.496114407	0.012586752	0.930867016	0.123797533	0.391698265
Monocytes	0.122657404	0.39610797	0.185893596	0.19617736	0.063654489	0.660540815
Macrophages M0	−0.460114755	0.000774047	0.335954176	0.017067361	−0.486332874	0.000342482
Macrophages M1	−0.555919473	2.77E‐05	0.553780282	3.02E‐05	−0.624084493	1.28E‐06
Macrophages M2	0.390417242	0.005062526	−0.521126216	0.000104357	0.505758741	0.000179196
Dendritic cells resting	0.130704262	0.365607297	−0.027473275	0.849789098	0.246847464	0.08395548
Dendritic cells activated	0.301761447	0.033193937	−0.210692781	0.14191842	0.376303131	0.007073781
Mast cells resting	0.419021201	0.002456036	−0.363413739	0.009484269	0.443981643	0.001239047
Mast cells activated	0.153314477	0.287792305	0.024107159	0.868018508	0.127131281	0.378970803
Eosinophils	−0.135437473	0.348349462	0.11214637	0.438110312	0.012877086	0.929276353
Neutrophils	−0.350851626	0.012485386	0.333611923	0.017904038	−0.427556733	0.001954912

### The downstream signaling pathways of MST1L, OLFM4, and DPP10 genes

**Fig. 6:**
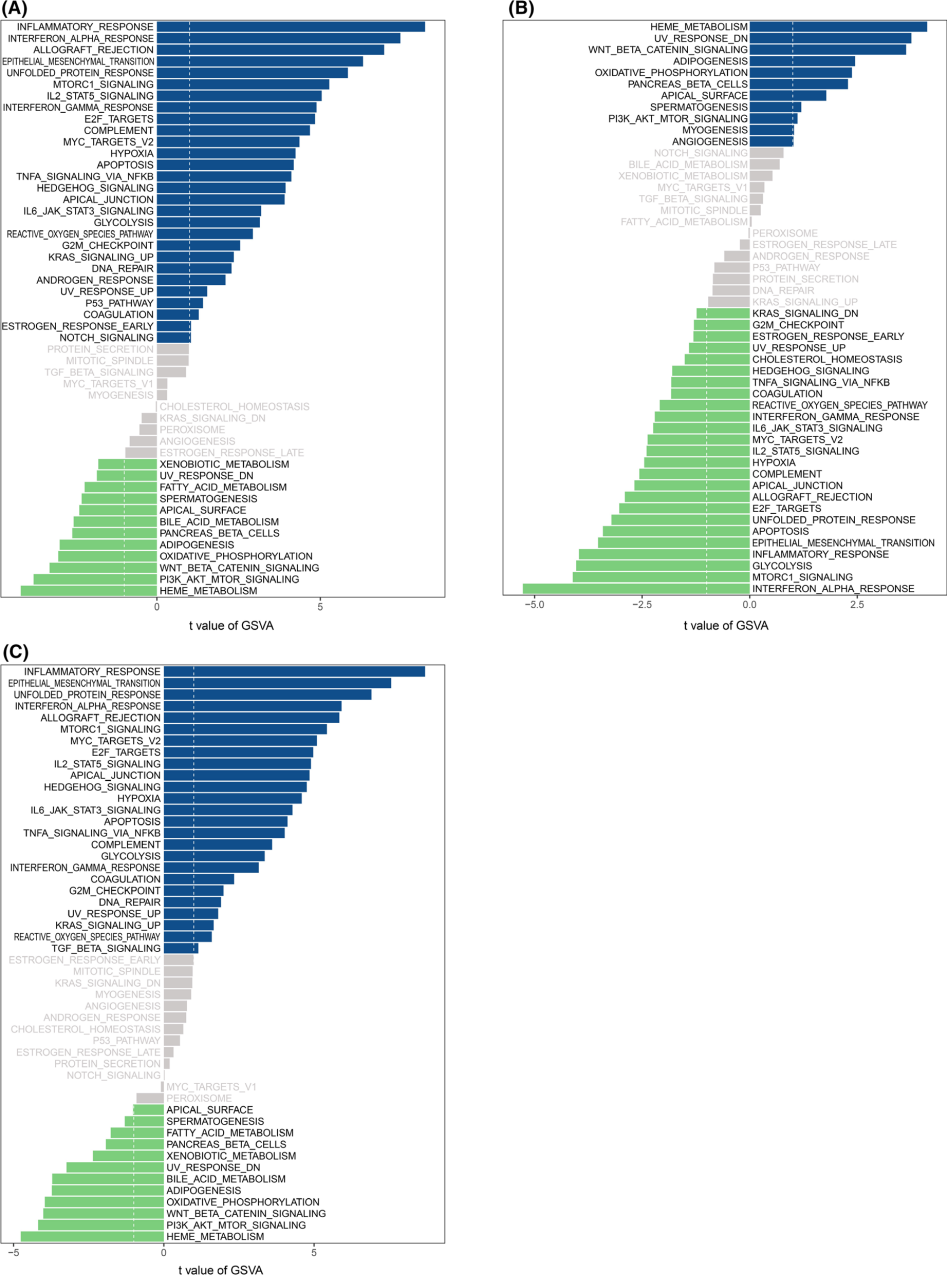
MST1L, OLFM4, and DPP10 were closely associated with WNT‐BETA‐CATENIN, PI3K‐AKT‐MTOR, and HEME‐metabolism signaling. (A–C) MST1L (A), OLFM4 (B), and DPP10 (C) were involved in WNT‐BETA‐CATENIN, PI3K‐AKT‐MTOR, and HEME‐metabolism signaling.

To identify the underlying role of these critical genes in the progression of ulcerative colitis, we determined the downstream signaling pathways by the GSVA ES. GSVA is an open‐source software package for R, which forms part of the Bioconductor project. In Fig. 6A–C, MST1L, OLFM4, and DPP10 were shown to be closely associated with WNT‐BETA‐CATENIN, PI3K‐AKT‐MTOR, and HEME‐metabolism signaling. The signaling pathway(s) required for these immune‐related biomarkers in the development of ulcerative colitis should be further verified in future studies.

### Identification of the mRNA expression of *SGK1*, *CEP55*, *ACSL1*, *OLFM4*, *MGP*, and *DPP10* in Raw264.7 cells and colon tissues of DSS‐induced colitis mice

**Fig. 7:**
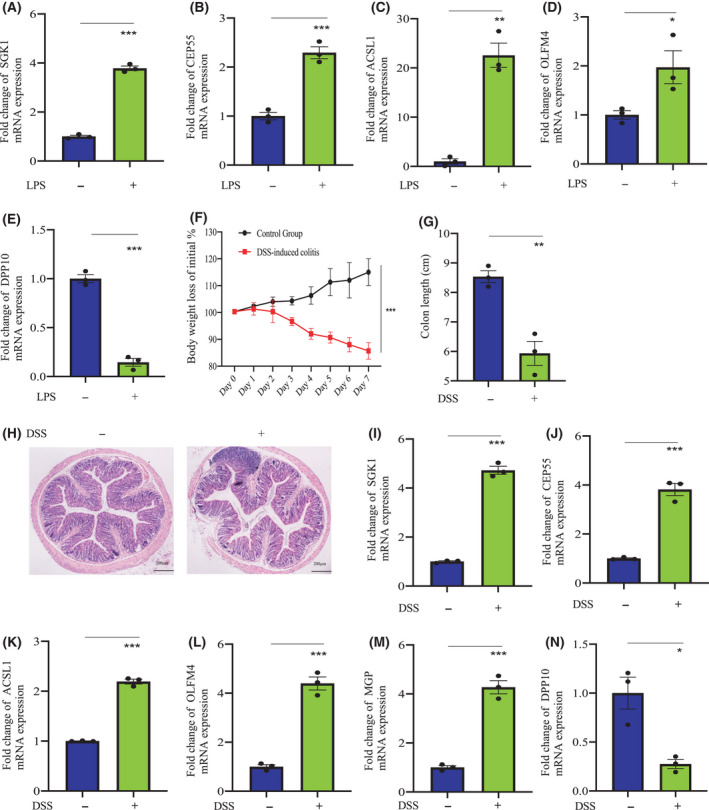
The mRNA expression of *SGK1*, *CEP55*, and *ACSL1* in Raw264.7 cells upon stimulation by LPS. (A–E) The mRNA level of *SGK1*, *CEP55*, *ACSL1*, *OLFM4*, and *DPP10* in Raw264.7 cells was changed by treatment with LPS. Raw264.7 cells (1 × 10^6^ cells) were plated onto 24‐well tissue culture plates and treated by 100 ng·mL^−1^ of LPS for 24 h, and cells were then harvested to determine the mRNA level by qPCR. (F–H) DSS‐induced colitis mouse model. (F) Bodyweight change (% of initial). (G) Length of the colon. (H) Hematoxylin and eosin staining of the colon (scale bar: 200 µm). (I–N) The mRNA level of *SGK1*, *CEP55*, *ACSL1*, *OLFM4*, *MGP*, and *DPP10* in the colon tissue of the DSS‐induced colitis mouse model was determined. Data (means ± SEM) shown (A–E: *n* = 3 independent experiments; G, I–N: *n* = 3 mice; two‐tailed Student's *t*‐test) were representative of three separate experiments. Compared with the indicated group, *P < 0.05, **P < 0.01, and ***P < 0.001.

To further validate the reliability of the computational approach in screening candidate biomarkers, we then examined the mRNA level of candidate biomarkers by biological studies. It has been reported that LPS induced macrophage inflammatory response [20]. To test the mRNA expression of *SGK1*, *CEP55*, and *ACSL1* in the context of inflammation, LPS was employed to induce inflammatory responses of macrophages. As shown in Fig. 7A–E, the mRNA expression of *SGK1*, *CEP55*, *ACSL1*, *OLFM4*, and *DPP10* in Raw264.7 cells was markedly upregulated by *in vitro* treatment with LPS. To further confirm the differential expression of these candidate genes between ulcerative colitis and normal groups, we determined the mRNA expression of *SGK1*, *CEP55*, *ACSL1*, *OLFM4*, *MGP*, and *DPP10* in colon tissues of DSS‐induced colitis mice (Fig. 7F–H). Compared with the normal group, the expression of *SGK1*, *CEP55*, *ACSL1*, *OLFM4*, and *MGP* was markedly increased in the DSS‐induced ulcerative colitis group. However, the expression of *DPP10* was strikingly decreased in the DSS‐induced ulcerative colitis group (Fig. 7I–N). These data were consistent with the results predicted by our bioinformatic methods.

## Discussion

Our study integrated two original microarray datasets, that is, GSE36807 and GSE65114. The analysis identified 216 DEMs, including 153 upregulated and 63 downregulated genes. The outcomes of GO and KEGG functional enrichment analysis indicated that these DEMs were shown to mainly be enriched in pathways associated with immune cell responses, such as neutrophil migration and regulation of leukocyte migration. These results indicated that immune responses play a crucial role in the development of ulcerative colitis. Indeed, previous studies have suggested that dysregulated genes in ulcerative colitis are primarily focused on the immune processes [21, 22] and excessive recruitment of activated neutrophils in the intestine results in mucosal injury and inflammation [23]. The rapid recruitment and inappropriate retention of leukocytes is a hallmark of all chronic inflammatory disorders, including ulcerative colitis and T cells at sites of inflammation [24]. Moreover, it has been clearly documented that CD4 T cells and NKT cells are a source of Th2‐ and Th17‐associated pro‐inflammatory cytokines, which contribute to intestinal mucosal inflammation [25]. In brief, these GO and KEGG analysis results provided new evidence to illustrate those immune processes play an essential role in the development of ulcerative colitis.

In addition, we identified eight candidate biomarkers, DPP10, MST1L, DPP10‐AS1, CEP55, ACSL1, MGP, OLFM4, and SGK1, by two independent algorithms, Lasso and SVM_RFE. These genes, especially MST1L, OLFM4, and DPP10, were validated in the GSE48958 dataset. MST1L is affiliated with the lncRNA class, and little is known about its function. MST1L is predicted to regulate macrophage chemotaxis by GO analysis. OLFM4, an extracellular matrix glycoprotein, facilitated cell adhesion [26]. It was initially cloned from human myeloblasts and selectively expressed in the inflamed colonic epithelium [27]. Dabiri et al. [28] reported that OLFM4 was associated with colorectal cancer. DPP10 is a vital regulator of Kv4‐mediated potassium channels, altering their expression and biophysical properties [29]. Allen et al. [30] reported that DPP10 cleaved the terminal dipeptides of cytokines and chemokines, consequently modulating inflammation. Furthermore, Park et al. found that DPP10 may play an important role in the development of colorectal cancer [31]. Hence, these candidate biomarkers, MST1L, OLFM4, and DPP10, may be involved in the development of ulcerative colitis. However, the *in vivo* effect of these genes on the progression of ulcerative colitis should be further investigated.

Since immune cells play an important role in ulcerative colitis pathogenesis, it is necessary to further study the relationship of these biomarkers to the immune response. CIBERSORT algorithm was used to assess the types of immune cells in ulcerative colitis. These candidate biomarkers, MST1L, OLFM4, and DPP10, were predicted to be strongly associated with immune cell infiltration of colitis, especially γδ T cells, neutrophils, and macrophages M1. However, these speculations require further study to verify the role of immune response by integrated regulation of these candidate genes in the progression of ulcerative colitis.

Taken together, candidate therapeutic biomarkers were selected from differentially expressed genes by Lasso and SVM_RFE, and then, the relationship between these candidate biomarkers and infiltrating immune cells was analyzed. Furthermore, the downstream signaling pathway implicated by these key biomarkers was predicted by GSVA. To further validate the expression of these candidate biomarkers in ulcerative colitis, we determined mRNA levels of *SGK1*, *CEP55*, *ACSL1*, *OLFM4*, and *DPP10* in LPS‐stimulated Raw264.7 cells by qPCR in *in vitro* bioassays. In addition, we also examined the expression of *SGK1*, *CEP55*, *ACSL1*, *OLFM4*, *DPP10*, and *MGP* in the colon tissues of DSS‐induced colitis mice. Consistent with the predicted computational results, the mRNA levels of these candidate genes were markedly changed in LPS‐stimulated Raw264.7 cells and inflamed colon tissues.

All in all, we identified eight candidate genes closely related to ulcerative colitis by machine learning algorithms. Of these genes, *SGK1*, *CEP55*, *ACSL1*, *OLFM4*, and *DPP10* were validated in both LPS‐stimulated Raw264.7 cells and inflamed colon tissues by qPCR. In addition, OLFM4 was only expressed in the ulcerative colitis samples and DPP10 was only found in the normal samples. It suggested that these genes may participate in the onset and/or progression of ulcerative colitis. Furthermore, more experiments in both *in vivo* and *in vitro* are needed to further clarify these findings in the future.

In addition, as reported by Gazouli et al. [32], the genome‐wide association study analysis showed that UC and CD have different subtypes. Then, it had different prognosis and therapeutic management for different disease subtypes. Biomarkers appeared as an important modality in IBD diagnosis, prognosis, and treatment [33]. Our findings indicated that several critical genes may act as diagnostic biomarkers of ulcerative colitis. However, it remains unknown whether these biomarkers can distinguish different endophenotypes. Hence, it is necessary to further investigate the distinguishing efficacy of these genes for different endophenotypes in the future.

## Conclusions

Our findings indicated that DPP10, MST1L, DPP10‐AS1, CEP55, ACSL1, MGP, OLFM4, and SGK1 may act as diagnostic biomarkers for ulcerative colitis and that differential immune infiltration cells may help to illustrate the progression of ulcerative colitis.

## Conflict of interest

The authors declare no conflict of interest.

## Author contributions

TH, KaW, PZ, and KeW designed the study. TH, KaW, and PZ participated in data acquisition and data analysis. TH, KaW, PZ, GZ, XY, YZ, ZZ, KZ, ZW, and KeW wrote the manuscript. All authors agree to the submission of the manuscript.

## Data Availability

The datasets of GSE36807, GSE65114, and GSE48958 can be obtained from GEO.
